# Two-dimensional electro-optical multiphoton microscopy

**DOI:** 10.1117/1.NPh.11.2.025005

**Published:** 2024-06-05

**Authors:** Deano M. Farinella, Samuel Stanek, Harishankar Jayakumar, Zachary L. Newman, Jacob Gable, James Leger, Aaron Kerlin

**Affiliations:** a University of Minnesota, Department of Neuroscience, Minneapolis, Minnesota, United States; b University of Minnesota, Department of Electrical and Computer Engineering, Minneapolis, Minnesota, United States

**Keywords:** multiphoton microscopy, electro-optical deflector, *in vivo* imaging, voltage imaging

## Abstract

**Significance:**

The development of genetically encoded fluorescent indicators of neural activity with millisecond dynamics has generated demand for ever faster two-photon (2P) imaging systems, but acoustic and mechanical beam scanning technologies are approaching fundamental limits. We demonstrate that potassium tantalate niobate (KTN) electro-optical deflectors (EODs), which are not subject to the same fundamental limits, are capable of ultrafast two-dimensional (2D) 2P imaging *in vivo*.

**Aim:**

To determine if KTN-EODs are suitable for 2P imaging, compatible with 2D scanning, and capable of ultrafast *in vivo* imaging of genetically encoded indicators with millisecond dynamics.

**Approach:**

The performance of a commercially available KTN-EOD was characterized across a range of drive frequencies and laser parameters relevant to *in vivo* 2P microscopy. A second KTN-EOD was incorporated into a dual-axis scan module, and the system was validated by imaging signals *in vivo* from ASAP3, a genetically encoded voltage indicator.

**Results:**

Optimal KTN-EOD deflection of laser light with a central wavelength of 960 nm was obtained up to the highest average powers and pulse intensities tested (power: 350 mW; pulse duration: 118 fs). Up to 32 resolvable spots per line at a 560 kHz line scan rate could be obtained with single-axis deflection. The complete dual-axis EO 2P microscope was capable of imaging a 13  μm by 13  μm field-of-view at over 10 kHz frame rate with ∼0.5  μm lateral resolution. We demonstrate *in vivo* imaging of neurons expressing ASAP3 with high temporal resolution.

**Conclusions:**

We demonstrate the suitability of KTN-EODs for ultrafast 2P cellular imaging *in vivo*, providing a foundation for future high-performance microscopes to incorporate emerging advances in KTN-based scanning technology.

## 1 Introduction

Signals in the brain are transmitted and transformed on a millisecond timescale. While these fast signals have long been studied with electrophysiology, precise spatial localization of these signals *in vivo* has primarily relied on two-photon (2P) imaging of fluorescent calcium indicators with far slower dynamics.^1^^–^^4^ Recently, impressive gains have been made in the development of genetically encoded indicators of neural activity with millisecond dynamics, including fast glutamate^5^ and voltage sensors.^6^^,^^7^ However, high fidelity recording of these reagents requires speed that is beyond conventional 2P microscopes, which typically utilize galvanometric mirrors and resonant mirrors, which are mechanically limited to 1 and 16 kHz line rates, respectively.^8^ To bypass these mechanical limits, methods based on acousto-optic effects have been developed, which have recently achieved line rates of up to 400 kHz^9^ but are ultimately limited by the transit time of acoustic waves in the crystal. In advanced scan systems that utilize passive optics to generate ultrafast scanning along one axis,^10^^–^^12^ the speed of other axes is still subject to fundamental inertial (acoustic or mechanical) limits or intentionally driven slowly to allow for accurate positioning. Furthermore, ideal multiphoton imaging systems for membrane-bound and ultrafast indicators should target excitation light to subcellular locations of interest. Targeted imaging^13^ can provide recordings with high signal-to-noise ratio (SNR), whereas dense raster scanning approaches can only achieve similar SNR via excitation with high average power (resulting in thermal damage) or high pulse energy (resulting in nonlinear photodamage). One approach to achieve high SNR and throughput is the simultaneous excitation of multiple foci;^6^^,^^14^^,^^15^ however, disentangling the emission from simultaneously excited sites can be challenging when imaging deep within tissue. None of the aforementioned technologies have the potential to achieve the sub-microsecond random access times that may eventually allow for sequentially interrogating thousands of targets deep within scattering tissue at kHz rates.

Electro-optical (EO) deflection has a fundamental response time on the order of femtoseconds.^16^ Most electro-optical deflectors (EODs) utilize the Pockels effect to deflect light at up to MHz rates, but these deflectors have a very limited field-of-view (FOV) when imaging with high numerical aperture (NA) objectives.^17^ This limited FOV is due to the low deflection efficiency of EODs utilizing the Pockels effect (∼1 to 10  μrad/V), owing to their small EO tensor coefficients (∼10  pm/V).^18^^,^^19^ The limitation ultimately arises in the form of dielectric breakdown, which occurs when driving with field strengths of above ∼kV/mm.^19^ A more useful figure of merit for a scanner than the total deflection angle is the number of resolvable spots it can produce. High performance commercial EOD systems utilizing the Pockels effect and kHz linear drivers (e.g., 400-120 Deflector and 302RM Amplifier, Conoptics Inc.) are limited to ∼15 resolvable spots [full width at half maximum (FWHM) criterion] in the near infrared (∼30 resolvable spots using custom kHz resonant drivers^17^).

**Fig. 1:**
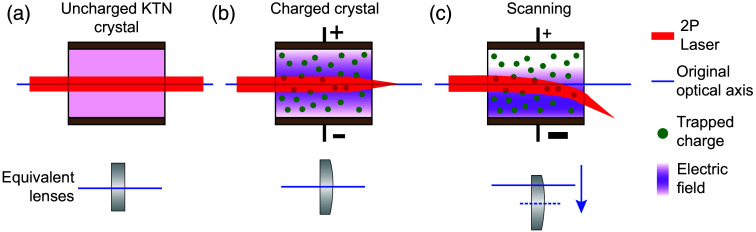
Principles of KTN-based laser deflection. (a) Prior to the application of DC voltage, the crystal contains no trapped charges and exhibits no lensing or scanning behavior. (b) When a DC voltage is applied to the crystal, electrons are injected into the crystal and become trapped. These trapped charges change the index of refraction in the crystal via the Kerr effect, effectively forming a lens inside the crystal. (c) When an AC drive voltage is applied to the charged crystal, the equivalent lens formed by the trapped charges shifts off the optical axis, resulting in scanning.

Since the 1960s, potassium tantalate niobate (KTN) has captured the attention of researchers, due to its potential for increasing the number of resolvable spots accessible to EODs into the 100s and beyond.^20^^–^^22^ Early attempts to utilize these crystals in beam scanners proved difficult due to challenges in crystal growth quality and strong temperature dependence to the EO coefficient.^19^ In the last decade, temperature-controlled EOD scanner modules utilizing high quality KTN crystals have become commercially available, which have achieved deflection efficiencies of ∼100  μrad/V by utilizing the Kerr EO effect (Fig. 1), rather than the Pockels effect. The availability of these crystals has reinvigorated interest in KTN scanners for a wide array of applications.^23^^–^^25^ Although there is debate about the physical mechanisms involved, KTN devices have been shown to have nanosecond response times, limited primarily by the speed of drive electronics.^26^^,^^27^

To investigate the suitability of KTN-based deflection technology for *in vivo* 2P imaging applications, we characterized the performance of the fastest commercially-available KTN EO deflector (KTN-EOD) under a range of illumination and drive parameters appropriate for 2P imaging of genetically encoded sensors. We then designed and implemented a two-dimensional (2D) EO 2P microscope and demonstrated *in vivo* imaging of membrane voltage at a frame rate of over 14 kHz. These results demonstrate the suitability of this technology for *in vivo* 2P imaging and provide a foundation for the future development of more powerful KTN-based deflectors and microscopes.

## 2 Methods

### 2.1 KTN-EOD Setup

A DC power supply (GPR-30H100, GW Instek) and linear amplifier (Trek 2100HF, Advanced Energy) were connected in series with the electrodes of the KTN-EOD (KSCHRM0850-00, NTT Advanced Technology Corporation) to provide a bias voltage and high-frequency drive voltage, respectively. A function generator (DDS Signal Generator, Koolertron) was connected to the input of the amplifier to provide the sinusoidal scanning waveform. The temperature of the KTN-EOD entrance facet was monitored with a thermal camera (A655SC, Teledyne FLIR). The thermoelectric cooler on the KTN-EOD was driven by a Peltier controller (TDC-1010A, Cell System). An integrated 405 nm LED illuminated the KTN-EOD crystal during scanning.

### 2.2 Stroboscopic Measurements of Resolvable Spots

This section describes the setup for the investigation of maximizing the speed of deflection (Sec. 3.2). A polarized beam from a 60 ps pulsed triggerable 970 nm semiconductor laser (EPL-980, Edinburgh Instruments) was spatially filtered by a 75  μm pinhole and then resized to form a round, collimated beam with a $1/e^{2}$ diameter of 0.44 mm. After rotating the polarization to align with the KTN-EOD scan axis, the KTN-EOD was placed at the beam waist. A function generator providing a sinusoidal drive waveform was also used to generate trigger signals for the laser source. The scanning beam from the KTN-EOD was collimated with a plano-concave cylindrical lens (f=−6.4  mm), and the scan range was collimated with a compound scan lens (f=71.4  mm) consisting of two spherical doublets (f=100  mm, f=250  mm) forming an intermediate image plane. The image plane of the scanning beam was then down-magnified 3× by an image relay consisting of two spherical achromatic doublets (f=150  mm, f=50  mm) to fit on a sCMOS camera sensor (CS165MU1, Thorlabs Inc.). By changing the phase between the trigger waveform and the sinusoidal scan, different scanning angles were interrogated. The total scan range was measured as the distance on the camera between the center of mass of focal spots located at the positive and negative extrema of the drive voltage range. For each drive voltage, two to five beam profiles were recorded, distributed across the scan range depending on the extent of the achieved range. The number of resolvable spots was calculated as the ratio of the total scan range to the average FWHM of a Gaussian fit to the beam profiles in the scan dimension.

The number of resolvable spots was measured at 200, 320, 440, and 560 kHz line rate using the deflector operating conditions recommended by the factory (temperature controller setpoint of 31.2 C, DC bias voltage of 218 V, and sinusoidal drive voltage amplitude of 270 $V_{\mathrm{pp}}$). In addition, the number of resolvable spots was measured at line rates from 200 to 1040 kHz in 120 kHz increments under optimized deflector operating conditions for each scanning frequency. To find the optimal operating conditions for each line rate, the DC bias voltage, Peltier set temperature, and drive voltage amplitude were iteratively adjusted to maximize the number of resolvable spots. In each case, the camera was translated along the optical axis to ensure that it was precisely positioned with the beam waist in the scanning dimension.

### 2.3 Influence of Laser Average Power on Performance

**Fig. 2:**
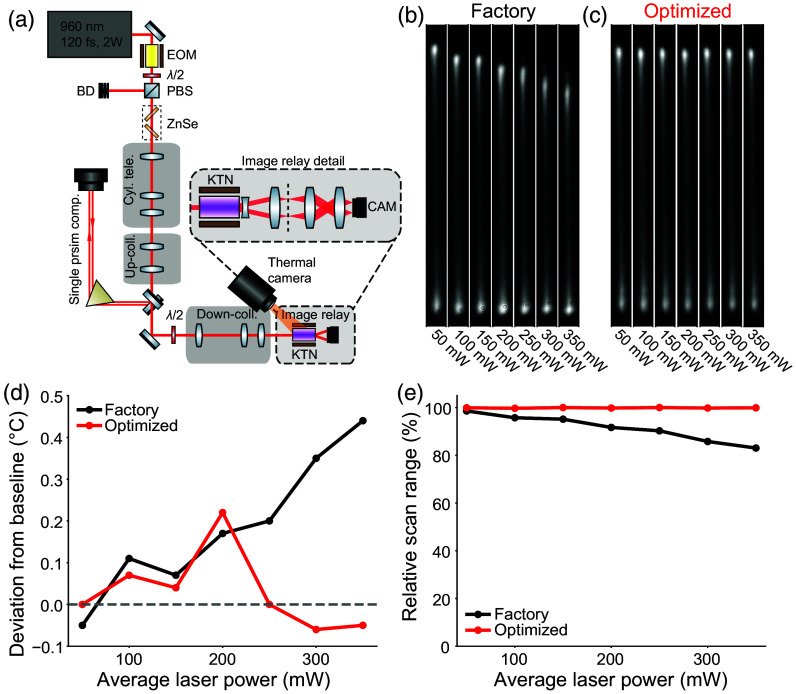
Stabilized deflection range across typical imaging powers for *in vivo* 2P imaging. (a) Experimental setup for capturing deflector scan profiles and thermal images, showing beam conditioning optics, KTN-EOD, and thermal camera. Inset shows optics used to relay the scanning beam to the camera. (b) Camera images of the scanning beam at each laser power using factory deflector settings. (c) Camera images of the scanning beam at each laser power using deflector settings manually optimized for each laser power. (d) Mean temperature deviation of the crystal at each laser power as measured from thermal imaging of the crystal face. (e) Relative scan range at each laser power compared to the 50 mW, optimized parameters case. EOM, EO modulator; λ/2, half-wave plate; BD, beam dump; PBS, polarizing beam splitter; CAM, camera; ZnSe, zinc selenide.

This section describes the setup for the investigation of the effects of scanning performance as a function of average laser power (Sec. 3.1). The beam from an 80 MHz pulsed laser source (InSight X3, Spectra-Physics) was circularized and resized to produce a round mode with a $1/e^{2}$ diameter of 0.47 mm using a series of spherical and cylindrical telescopes as shown in Fig. 2(a). Based on our previous experience balancing excitation efficiency with scatter length during *in vivo* imaging of GFP variants,^28^^,^^29^ we chose to study deflector performance at a central wavelength of 960 nm. The laser pulses were compressed using the DeepSee internal compression offered by the Insight X3 in conjunction with a home-built single prism (H-ZF72A, United Crystals) compressor.^30^ Compression was verified by tight-focusing the beam onto a fluorescent slide in the KTN plane and maximizing fluorescence intensity by varying prism separation using the single prism compressor. After verification of compression, the KTN-EOD was placed at the beam waist. An image of the scan range was relayed to the surface of a sCMOS sensor to monitor the beam profile (see Sec. 2.2 for details).

For each scan profile measurement, the KTN-EOD was temperature-controlled and was provided with a DC bias and AC drive voltage. The beam was initially blocked, and a thermal camera image of the KTN crystal face was captured, serving as a baseline temperature measurement. The incident laser power was adjusted using a combination of a half-wave plate and PBS power control module, as well as a Pockels cell (350-80, Conoptics Inc.). The power was measured at the KTN plane before each measurement with an optical power meter (S415C, Thorlabs Inc.). The beam was then unblocked and allowed to transit the crystal while the KTN-EOD was scanning. After 1 min, an image of the scanning beam and a thermal camera image were captured. A set of measurements were performed using factory recommended deflector operating conditions (see Sec. 2.2) at laser powers of 50 to 350 mW in increments of 50 mW. These measurements were repeated for the same laser powers, with a corresponding reduction in the temperature controller set point for each measurement. The new temperature controller set point for each laser power was selected to achieve a scan range equivalent to the scan range observed at low laser power (50 mW).

### 2.4 Influence of Laser Peak Intensity on Performance

This section describes the setup for the investigation of the effects of scanning performance as a function of laser peak intensity (Sec. 3.1). Images of the scanning beam were captured at a low power (10 mW) and a high power (350 mW) in the same experimental setup as in Sec. 2.3. In this instance, the $1/e^{2}$ beam diameter at the entrance aperture of the KTN-EOD was measured to be ∼0.45  mm. At both low power and high power, scan range data were measured using compressed and chirped pulses. The compressed laser pulse duration was determined by minimizing the pulse duration after the KTN via manipulation of our single prism compressor (see Sec. 2.3) and was measured directly via second harmonic generation frequency-resolved optical gating (SHG-FROG^30^^–^^33^) to be 118 fs. Chirped pulses were created through the inclusion of 3 sets of two 5 mm uncoated zinc selenide (ZnSe) windows (WG71050, Thorlabs Inc.) in Brewster pairs (to compensate lateral Snell shifts and minimize reflective losses). Using the measured pulse spectrum and phase as the input, the theoretical group delay dispersion (GDD) and third-order dispersion from the ZnSe windows were added to simulate the resulting chirped pulse duration using PyNLO^34^^,^^35^ to be ∼565  fs. Due to reflective losses from the addition of the ZnSe windows, the highest available power for the chirped pulses was reduced to 335 mW.

### 2.5 2D KTN-EOD Relay Module

To incorporate the KTN-EOD into our custom relay module, the small plano-concave cylindrical lens affixed near the exit aperture by the manufacturer was removed. The relay module shown in Fig. 5 collimates the scanning beam of KTN-EOD 1 in the x-dimension and relays a conjugate plane downstream using a compound scan lens (x-conjugator) consisting of three NIR-coated f=50  mm spherical achromatic doublets (45-803, Edmund Optics). The scanning beam (which begins focusing in the y-dimension after the scan lens of KTN-EOD 1) is then collimated with an f=19  mm cylindrical plano-convex singlet (y-collimator) (CKX019AR.16, Newport Corporation). After a 90 deg polarization rotation via half-wave plate (WPHSM05-980, Thorlabs Inc.), KTN-EOD 2 is placed at the conjugate plane of KTN-EOD 1 with electrodes oriented orthogonally to KTN-EOD 1. The same lenses rotated by 90 deg were positioned after KTN-EOD 2 in the same arrangement to create a second conjugate plane common to each KTN-EOD at the exit of the scanner relay module. Fine positioning of the KTN-EODs was enabled by a triple-axis micrometer translation stage (PT3, Thorlabs Inc.).

### 2.6 Measurement of 2P Point-Spread Function

Laser light (960 nm, InsightX3) was delivered to a single-prism compressor (PBH71) to compensate for excess GDD beyond the compensation offered by the internal compressor of the ultrafast laser. The KTN-EOD scanner relay module and a redundant set of galvanometer mirrors (6215H, Cambridge Technology) were incorporated into a homebuilt 2P microscope (MIMMS 2.0 design; Janelia Research Campus, HHMI). An up-collimating telescope was used prior to the compressor to enlarge the Rayleigh range and ensure a collimated beam throughout the pulse compressor. The beam was then down-collimated and reshaped through a series of three telescopes before entering the KTN-EOD relay module (see Sec. 2.5) with a round collimated beam size of 0.5 mm $1/e^{2}$ diameter. Following the KTN-EOD relay module, the scanning beam was enlarged and scan range conjugated to a plane between the galvanometer mirrors through a 5× magnification relay. The two compound relay lenses consisted of two f=150  mm spherical achromatic doublets (49-362, Edmund Optics) and two f=750  mm spherical achromatic doublets (67-336, Edmund Optics), respectively. A compound scan lens consisting of three spherical achromatic doublets f=150  mm (AC508-150-B Thorlabs) and Nikon tube lens (f=200  mm) form a final 4× afocal relay to the back-pupil of the 25× objective (XLPLN25XWMP2, Olympus), ultimately resulting in an effective NA of ∼0.8. Epi-detected fluorescence signal photons were reflected into a detection arm by a long-pass primary dichroic (FF705-Di01, Semrock) and further filtered by a band-pass emission filter (FF01-520/70, Semrock) before being detected by a photomultiplier tube (PMT2101, Thorlabs).

Fluorescent beads with a 0.2  μm diameter (F8811, ThermoFisher Scientific) were used to measure the point-spread function and FOV of the 2D KTN-EOD microscope (Fig. 6). A z-stack of images with a 13  μm by 13  μm FOV (177 pixels by 40 lines) was acquired with KTN-EOD line rate of 560 kHz using ScanImage (MBF Bioscience). A motorized z-stage (PLS-X, Thorlabs Inc.) was used to manually translate the focal plane in 2  μm increments between each image acquisition. Image stacks were processed using custom Python scripts. Briefly, bidirectional scanning offsets were corrected in post processing to ensure correct registration of the 2D scanning. Then, 10,000 frames were averaged to generate a single image for each z-position. The average projection images for all z-positions were then scaled (using bicubic interpolation) to generate isometric 3D voxels (0.068  μm). Fluorescence profiles were extracted using this 3D volume along the respective axes with the FWHM being calculated directly from the profiles.

### 2.7 *In Vivo* Voltage Imaging

All animal procedures were carried out in accordance with protocols approved by the University of Minnesota Animal Care and Use Committee. A circular (2.5 mm diameter) craniotomy was made above the primary visual cortex (V1; 0.5 mm anterior, 2.7 mm lateral of lambda, 400  μm deep) of a C57BL6 mouse. Cortex was injected with 150 nL AAV1-EF1a-DIO-ASAP3 ($5\times 10^{12}\;\text{titer}$; 132318, Addgene) and AAV9-CamKII-Cre ($2.1\times 10^{9}\;\text{titer}$; 105558, Addgene) to sparsely label layer 2/3 neurons with ASAP3. A window (triple #1 coverglass 2.5/2.5/3.5 mm diameter) was fixed to the skull using dental adhesive. A head fixation metal bar (with a metal loop surrounding the window) was also implanted posterior to the window using dental acrylic. Imaging of spontaneous activity was performed on an awake and head-fixed mouse. A reference image was taken of a neuron using the redundant galvanometer mirrors (158  μm by 158  μm FOV) at a frame rate of 1.7 Hz. High-speed imaging was then performed using the KTN-EODs (Fig. 6). For the *in vivo* experiment, the 5× magnifying relay in Fig. 6 (RL3 and RL4) was replaced with a 2.44× magnifying relay to produce an FOV of 27  μm by 10  μm (64 pixels by 32 lines), at the cost of reducing the effective to NA∼0.39 (estimated lateral FWHM: 0.93  μm; estimated axial FWHM: 10.3  μm). The first compound relay lens (RL3) consisted of f=750  mm (Edmund Optics, 67-335) and f=100  mm (49-360, Edmund Optics) spherical achromatic doublets. The second compound relay lens (RL4) consisted of f=750  mm (67-335, Edmund Optics) and f=300  mm (AC254-300-B, Thorlabs Inc.) spherical achromatic doublets. The cell membrane of the soma was scanned with 960 nm light with intermittent short continuous imaging bouts at either 14.7 kHz for 1 s or 15.6 kHz for 0.9 s repeated every 5 s. Power at the sample during imaging bouts was 39 mW. The minor differences in frame rate from session to session reflected instability in the number of lines the microscope control software (ScanImage, MBF Bioscience) allotted for flyback. Images were processed using custom MATLAB and Python scripts to extract time courses. Images were motion corrected using cross-correlation^36^ and regions were drawn manually over the cell membrane to extract ASAP3 fluorescence traces from three separate neuron somas recorded in one mouse. $\Delta F/F_{0}$ traces were calculated using an average of the first 50 ms of each imaging trial as the $F_{0}$ fluorescence value. Estimates of frame-to-frame $\Delta F/F_{0}$ noise (σ) for each neuron were calculated from the average of the spectral density between 0.25 and 0.5 times the Nyquist frequency.^37^ Equivalent $\Delta F/F_{0}$ noise at 1 kHz was calculated by assuming averaging of Poisson noise such that $\sigma_{1\;\mathrm{kHz}}=\sigma_{\mathrm{fr}}/\surd (\mathrm{fr})$, where fr is the imaging frame rate in kHz. To calculate bleaching time constants, trial traces were first normalized by dividing by the average of the first 20 frames of each trial. Decay time constants (τ) were then estimated by least-squares fitting of a single exponential decay model to the trial-averaged normalized trace. Normalized signal after 1 s of imaging was estimated from the decay time constant of each neuron.

## 3 Results

### 3.1 Deflection of Femtosecond Infrared Pulses with Average and Peak Energies Typical of *In Vivo* 2P Imaging

In the context of *in vivo* 2P laser scanning, the KTN-EOD is subject to higher laser average powers than the specimen due to losses from downstream optics. Therefore, we investigated the performance of the KTN-EOD at a range of laser average powers extending above *in vivo* limits (up to 350 mW) [Fig. 2(a)]. As laser power was increased, the scan range of the KTN-EOD decreased and the beam profile became distorted as shown in Figs. 2(b) and 2(e). However, by reducing the set temperature on the Peltier temperature controller (see Table S1 in the Supplementary Material), we were able to recover the lost range [Figs. 2(c) and 2(e)]. Thermal camera measurements indicated that at factory-defined controller settings, increases in laser power result in uncompensated heating of the KTN crystal [Fig. 2(d)]. Optimization of the temperature controller settings reduced the observed heating, especially at the highest tested laser powers [Fig. 2(d)].

**Fig. 3:**
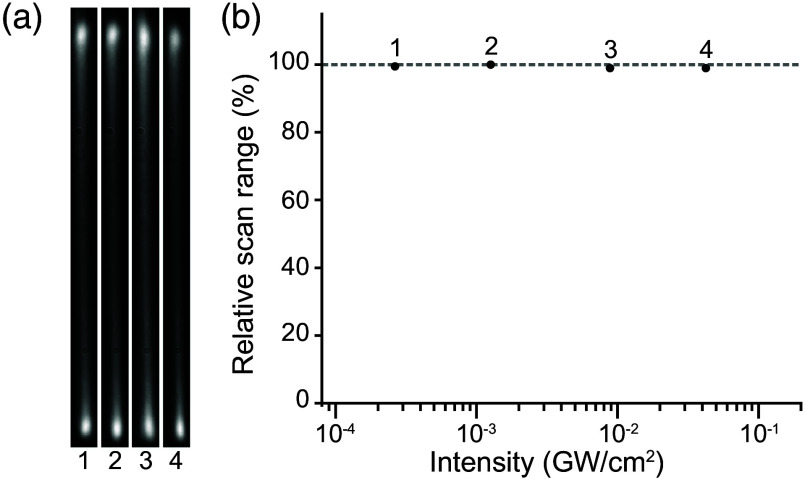
Intensity dependent deflector performance at a range of average powers at or above those typical for *in vivo* 2P imaging. (a) Beam profiles observed at different laser powers and pulse durations. (b) Scan range percentage for each combination of pulse duration and laser power (case 1: 10 mW, 565 fs; case 2: 10 mW, 118 fs; case 3: 335 mW, 565 fs; case 4: 350 mW, 118 fs) relative to 10 mW, 118 fs (case 2). Horizontal axis shows the calculated optical peak intensity for each case.

It has been reported that ultrashort pulses can cause beam profile deformation in KTN-based scanners.^23^ To verify that this deflector was suitable for *in vivo* 2P imaging, we performed deflection tests using femtosecond infrared laser pulses (see Secs. 2.3 and 2.4) across a range of peak intensities [Fig. 3(b)]. We measured the deflector scan range at high and low laser powers for both compressed pulses and positively chirped pulses, respectively (see Sec. 2.4). Our laser pulses were compressible to ∼118  fs FWHM using our external prism compressor. Positively chirped pulses were achieved by the insertion of bulk ZnSe, which broadened the pulses to ∼565  fs (see Sec. 2.4). These pulse durations (along with the measured power and known rep-rate) were then used to approximate the peak intensity of the laser pulses [Fig. 3(b)]. We observed that although increasing average laser power was seen to reduce the scan range (which could then be recovered by temperature controller optimization), peak intensity did not have a direct effect on deflector performance (Fig. 3). When holding average power nearly constant and changing the peak intensity by a factor of 4.8 we saw no significant change in the performance. These results suggest that when filling the aperture of the KTN-EOD, ultrafast nonlinearities of KTN are negligible at pulse parameters typical for *in vivo* 2P imaging.

### 3.2 Maximizing the Speed of Deflection

**Fig. 4:**
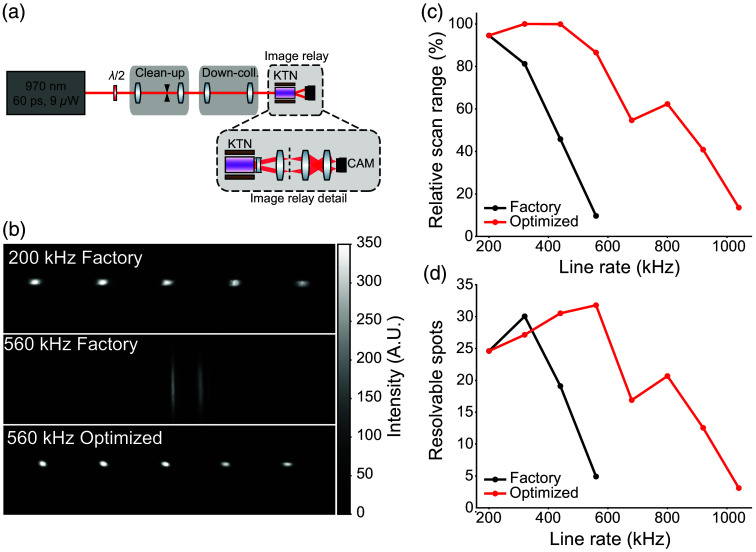
Deflector performance at line rates exceeding manufacturer specification. (a) Experimental setup for measuring the number of resolvable spots at different line rates. Inset shows optics used to relay the scanning beam to the camera. (b) Camera images of the scanning beam demonstrating how optimization of deflector parameters can recover performance at line rates exceeding the manufacturer specifications. (c) Relative scan range achieved for each line rate with factory deflector operating parameters and manually optimized parameters. (d) Number of resolvable spots provided by the deflector for each line rate with factory deflector operating parameters and manually optimized parameters.

We next characterized the deflector performance across a range of line rates using a triggerable pulsed laser and camera [Fig. 4(a)]. The triggerable source enabled us to make stroboscopic measurements of scan range and estimate the number of resolvable spots. The KTN-EOD was designed to operate at a line rate of 200 kHz, but we found that it could be operated at higher rates. Using factory recommended operating parameters, performance degraded as the line rate was increased above specification [Figs. 4(b)–4(d)]. However, it was possible to recover performance for a range of higher line rates by tuning the operating parameters (see Sec. 2.2 and Table S2 in the Supplementary Material). The deflector provided up to 32 resolvable spots at a line rate of 560 kHz with optimized operating parameters. Thermal measurements revealed that when driving beyond 560 kHz, a pronounced temperature gradient across the crystal appears, after which the performance cannot be recovered by control parameter optimization (Fig. S1 in the Supplementary Material). Due to the strong temperature dependence of the relative permittivity,^38^ a more sophisticated thermal management strategy will likely be needed to operate reliably at higher frequencies.

### 3.3 Design and Implementation of a 2D EO 2P Microscope

**Fig. 5:**
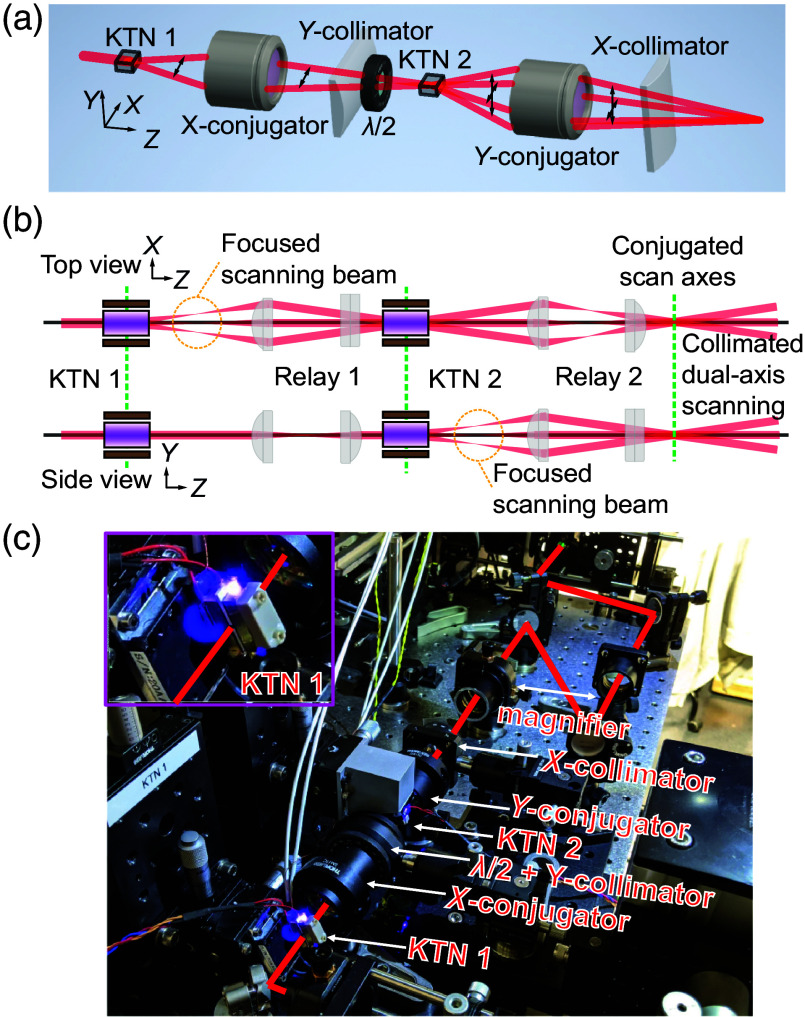
Design and implementation of a 2D KTN-EOD relay module. (a) Design overview of 2D KTN-EOD relay module with conjugated, orthogonally oriented KTN-EODs. Black arrows indicate laser scanning direction. λ/2, half-wave plate. (b) Projected views of beam propagation in the relay system. The first lens in the relay conjugates the scanning pivot points of both the KTNs. The second lens (cylindrical) collimates to make it a circular scanning beam. Dashed green lines are conjugate planes. (c) Photograph of the prototype microscope. Upper left inset: magnified image of first deflector during LED illumination. Superimposed red line illustrates the excitation path.

As opposed to conventional laser scanning technologies that produce a scanning collimated beam, the KTN-EOD produces a scanning beam that is strongly focused in the scanning dimension [Figs. 1(a)–1(c)]. A 2D KTN-EOD relay module compensates for the uniaxial focusing of the first scanner to generate a collimated beam of the appropriate size at the principal plane of the second scanner. As shown in Fig. 5, the scanning beam from KTN-EOD 1 was collimated in the x-dimension and relayed to KTN-EOD 2 with a compound scan lens (x-conjugator). The scanning beam that is now focused in the y-dimension was then collimated with a cylindrical plano-convex singlet (y-collimator). After a 90 deg polarization rotation via half-wave plate, KTN-EOD 2 was placed at the conjugate plane of KTN-EOD 1 with electrodes oriented orthogonally to KTN-EOD 1. An identical set of lenses rotated by 90 deg were positioned after KTN-EOD 2 to conjugate the scanning in the y-dimension and collimate the scanning beam in the x-dimension to a common conjugate plane at the exit of the scanner relay module.

**Fig. 6:**
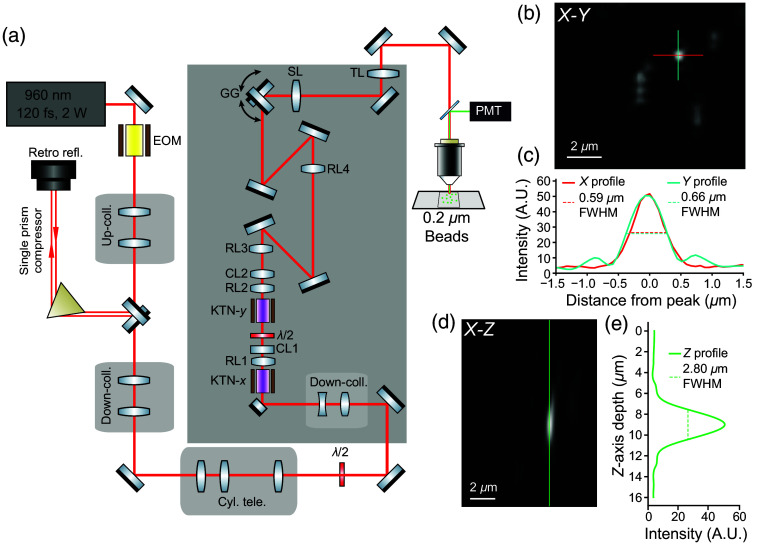
Implementation and optical performance characterization of a 2D EO 2P microscope. (a) Layout of complete optical path including light source, beam conditioning optics, scanners, optical relays, and detection scheme. (b) Image of a single X−Y plane of 0.2  μm diameter fluorescent beads when imaging via the 2D KTN-EOD scanners. (c) Vertical (cyan) and horizontal (red) line-outs of the intensity profile and FWHM determination of a selected bead. (d) Image of an X−Z slice of the bead, and (e) axial (green) line-out of the intensity profile and FWHM determination. EOM, EO modulator; λ/2, half-wave plate; KTN-X/KTN-Y, KTN-EOD crystals; RL1, x-conjugator; CL1, y-collimator; RL2, y-conjugator; CL2, x-collimator; RL3/RL4, relay lenses; GG, galvanometer scanning mirror pair; SL, scan lens; TL, tube lens; PMT, photomultiplier tube.

The 2D KTN-EOD microscope required several peripheral beam conditioning telescopes, as shown in Fig. 6(a) (see Secs. 2.3 and 2.6). A peripheral single-prism pulse compressor was required in addition to the internal compensation of the ultrafast laser (see Sec. 2.6) to accommodate the GDD of the 2D KTN-EOD scanner relay module. The GDD of the KTN crystal was approximated in its operating mode by maximizing second harmonic generation signal from a beta barium borate crystal before and after its introduction to the path via the manipulation of prism separation. Based on the difference in separation, the predicted difference in compensated GDD required to re-compress the pulses was $∼|4500\;{\mathrm{fs}}^{2}|$, providing an estimate for the KTN.^39^ Accounting for the GDD of the other scan optics from their Sellmeier curves, the total GDD of the 2D-KTN-EOD scanner relay was $∼15,000\;{\mathrm{fs}}^{2}$.^35^ An up-collimating telescope was used to enlarge the Rayleigh range during transit through the single prism compressor, and a three-lens cylindrical telescope and down-collimating telescope were used to correct for astigmatism and place the beam waist downstream. Due to the relatively short Rayleigh range of a 0.5 mm $1/e^{2}$ diameter beam, a final down-collimating telescope was required immediately before the 2D KTN-EOD relay module to ensure that the aperture was filled with a collimated beam, where a half-wave plate ensures that the polarization axis of the laser is aligned to the scan-axis of the KTN-EOD. Following the 2D KTN-EOD relay module, the 2D scanning beam was magnified by 5×, with its conjugate plane relayed onto a set of redundant galvanometer mirrors. This redundant set of scanners allowed for imaging of larger FOVs to identify targets of interest to interrogate with the smaller FOV afforded by the KTN-EOD scanning. The conjugate plane at the galvanometer mirror pair was then relayed one final time to the back aperture of our objective, underfilling to an approximate NA of 0.8.

A z-stack of images was acquired from a fluorescent bead sample (0.2  μm diameter) to measure the PSF and FOV of the 2D-EOD microscope (see Sec. 2.6). The FOV was measured to be 13  μm×13  μm, and the lateral PSF FWHM was found to be 0.59 and 0.66  μm in the horizontal and vertical dimensions, respectively [Figs. 6(b) and 6(c)]. Along the optical axis, the z-PSF was found to have an FWHM of 2.8  μm [Figs. 6(d) and 6(e)]. The deviation of the measured focal volume from that of a diffraction limited 2P focal volume at 0.8 NA (lateral FWHM: 0.45  μm; axial FWHM: 2.25  μm) is likely due in large part to imperfections in the compact optical relays built from off-the-shelf components.

### 3.4 Validation: *In Vivo* 2P Imaging of a Voltage Indicator at >14 kHz

**Fig. 7:**
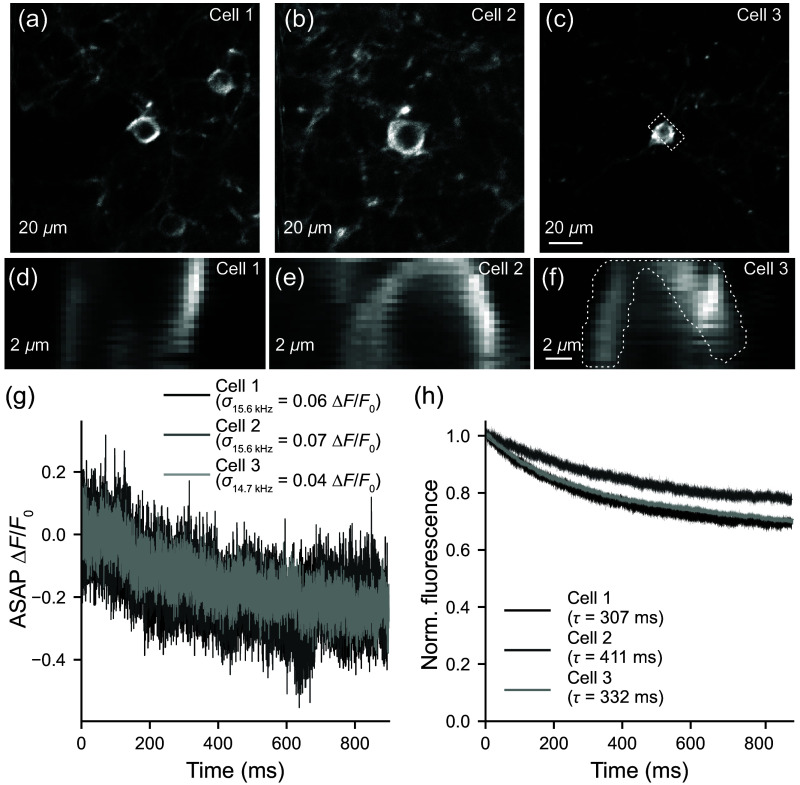
*In vivo* characterization of 2D EO microscope bleaching kinetics. (a)–(c) 2P images of three layer 2/3 pyramidal neurons expressing ASAP3 collected using standard scanning galvanometers. (d)–(f) 2D KTN-EOD scanning images (mean of 1000 frames) of the regions marked by dashed boxes in (a)–(c). Images were acquired at both [(d),(e)] 15.6 kHz and (f) 14.7 kHz frame rates. (g) Example single trials showing ASAP bleaching $\Delta F/F_{0}$ for the three cells membrane regions indicated with the dotted lines in (d)–(f). SNRs (σ) are indicated in the corresponding legends. (h) Trial-averaged normalized fluorescence exponential decay bleaching kinetics (shading indicates mean ± standard error of the mean) were calculated for all three cells (cell 1 N=106 trials; cell 2 N=118 trials; cell 3 N=117 trials; N=1 mouse). Exponential decay fits to the trial averaged data are shown with the solid lines and time constants (τ) are provided for each of the three cells in the respective legends. The objective back-aperture was underfilled to an effective NA of ∼0.39 (with a theoretical PSF of lateral FWHM: 0.93  μm; axial FWHM: 10.3  μm).

To test the ability of the 2D KTN-EOD microscope to conduct cellular resolution 2P imaging *in vivo*, we expressed a 2P-compatible genetically encoded voltage indicator (${\mathrm{ASAP}3}^{6}$) in layer 2/3 neurons of the primary visual cortex (V1) of adult mice. Beam magnification after the KTN-EOD relay module was reduced, which resulted in an effective NA of ∼0.39 (corresponding to expected PSF of lateral FWHM: 0.93  μm; axial FWHM: 10.3  μm) to obtain a larger x-axis FOV (see Sec. 2.7). Neurons expressing ASAP3 were identified in head-fixed mice using standard 2D galvanometer imaging [Figs. 7(a)–7(c)] and then imaged at either 14.7 kHz or 15.6 kHz frame rates using the 2D KTN-EOD light path [Figs. 7(d)–7(f)]. To assess ASAP3 signal quality and bleaching rate during 2D KTN-EOD imaging, imaging was carried out in 0.9 to 1.0 s bouts interleaved with 4.0 to 4.1 s dark periods that allowed the signal to recover from bleaching. High frequency noise during imaging bouts was 0.058±0.007 $\Delta F/F_{0}$ and signals decayed with a bleaching time constant of 0.35±0.03  s [Figs. 7(g) and 7(h)]. For comparison with another ultrafast scanning approach,^10^ we estimated (see Sec. 2.7) the equivalent noise after averaging down to 1 kHz sampling (0.015±0.002 $\Delta F/F_{0}$) and the normalized signal after 1 s of imaging (0.74±0.02). The estimates indicate that net emission and bleaching during 2D KTN-EOD imaging of ASAP3 were not far from those obtained using other kHz scanning approaches.^10^ Due to the fact that the imaging FOV was small relative to brain motion, the correction of the signal for motion artifacts was particularly challenging and sparse deflections in the ASAP3 signal could not be confidently classified as either artifacts or action potentials. Nonetheless, these results demonstrate that the 2D KTN-EOD microscope was capable of cellular imaging *in vivo* with high spatial and temporal resolution.

## 4 Discussion and Conclusion

This work demonstrates that KTN-EODs are capable of >10  kHz frame rate 2P cellular imaging *in vivo*. Concerns regarding the impact of nonlinear absorption on beam quality had discouraged the investigation of KTN-EODs for multiphoton imaging. Previous work evaluating the suitability of KTN-EODs for micromachining found that ultrafast (400 fs) infrared laser pulses generated substantial nonlinear beam deformation.^23^ Beam deformation due to any nonlinear optical phenomena would complicate the integration of these scanners into multiphoton microscopes by generating aberrations that are dependent on pulse intensity. The contribution of different nonlinear $\chi^{\left(3\right)}$ strong-field phenomena (e.g., Kerr lens induced self-focusing) to KTN lensing is poorly understood, so beam deformation cannot be easily predicted from first principles. Nonetheless, we noted that studies of KTN-EOD suitability for micromachining^23^ utilized μJ pulse energies and observed deformation at a fluence of $∼10\;\mathrm{mJ}/{\mathrm{cm}}^{2}$, far higher than the fluence that the nJ pulses of a typical laser for 2P imaging would generate in the KTN-EOD. Our results demonstrate that at the typical pulse duration (∼120  fs) and nJ pulse energies delivered to 2P imaging scanners, ultrafast nonlinearities are negligible. In approaches where higher energy pulses are typically used (e.g., low rep-rate, multifocal), a broader pulse bandwidth could be used to mitigate unwanted nonlinear effects inside the KTN. The GDD pre-compensation necessary for efficient imaging with broader bandwidth pulses would produce longer pulse durations outside of the focal plane than those that occur when compensating the dispersion of narrower bandwidths. This behavior could be exploited to ensure that laser pulses have sufficiently low peak intensity during propagation through the KTN-EOD.

We observed that the deflection range of the KTN-EOD decreased with increasing average power of incident light, as well as with increasing line rate. We did not attempt to determine the precise physical phenomena responsible for these reductions in deflection. However, our thermal imaging confirmed that at high average power or high line rate, the KTN-EOD temperature control system was unable to maintain a stable and uniform temperature across the crystal when using factory recommended settings. Adjusting the temperature control system largely eliminated these reductions for powers up to 350 mW and line rates up to 560 kHz. Thus, considering the temperature-dependence of the permittivity of KTN,^38^ we suspect that improvements in the thermal design of KTN-EODs could reduce the sensitivity of these devices to average power and drive frequency, though we cannot rule out the possibility that non-thermal phenomena (e.g., trapped-charge dynamics^23^) also contribute to the deflector sensitivity to these factors.

To our knowledge, no previous study has implemented a fully EO, 2D 2P microscope. EODs utilizing the Pockels effect have been previously incorporated into stimulated emission depletion imaging systems for fast scanning along one axis^17^ or pixel hopping.^40^ However, the small acceptance angle of commercial Pockels-based EODs makes it challenging to relay two EODs for dual-axis scanning. Our dual-axis KTN-EOD relay design is similar to previous one-photon designs^41^ implemented in a commercially available handheld device (2D KTN Optical Scanner, NTT-AT Corp.). However, our design was optimized for high performance scanning of near-IR wavelengths and can be easily adjusted to changes in deflector focusing under different drive conditions.

Line scan rates up to 4 MHz with 80 resolvable spots per line have been demonstrated using passive, cavity-based scanning methods;^10^^–^^12^ however, the utilization of a galvanometer for the slow axes limits frame rates to a maximum of 3 kHz. Recently, Li and colleagues^9^ developed a high bandwidth AOD system capable of scanning ∼53 2P resolvable spots at 400 kHz and frame rates up to 10 kHz (40 lines). We found that a single KTN-EOD was capable of scanning 32 one-photon resolvable spots of 960 nm light at 560 kHz. However, our fully integrated 2P system only obtained 23 spots per line, likely due to residual aberrations and vignetting introduced by our inexpensive relay system that incorporated only stock lenses. With custom relay optics, we expect the performance of an EO 2P microscope based upon the commercially available KTN-EOD to meet or exceed the performance of the fastest AOD scanning systems.

2P imaging of a small FOV at kHz rates will be critical for many measurements in neuroscience, including measurement of compartmental voltage interactions in dendrites, glutamate dynamics at the synaptic cleft, and blood velocity *in vivo*. However, broad adoption of EO 2P microscopy will require the development of EOD technology with more resolvable spots (enabling a larger FOV) and random-access capabilities (enabling higher SNR recordings). Large aperture KTN-EODs that would provide a far greater number of resolvable spots are the focus of ongoing research^42^ geared toward achieving homogeneous, high density charging of KTN across a large electrode gap. Other research avoids the challenges of achieving high-density charging of large KTN crystals by thermally engineering the polarization inside of the crystal to optimize deflection under conditions of low density charge.^43^ Furthermore, recent work has demonstrated step deflections of a few nanoseconds when KTN crystals are driven with ultrafast electronics,^24^ suggesting they are capable of sub-microsecond random access, which is impossible with cavity-based scanning or AOD systems. The potential of KTN-based technology to enable kHz interrogation of neural activity is just beginning to be explored, and the performance of this microscope should be considered a lower bound on what can be achieved.

## Supplementary Material



## Data Availability

All related data, analysis code, and design files are publicly accessible at https://github.com/kerlin-lab/Farinella_2024.
