# Supplementary material for: Two-dimensional electro-optical multiphoton microscopy
Source: Neurophotonics. 2024 Jun 5;11(2):025005. doi: 10.1117/1.NPh.11.2.025005 (PMC11151658; doi:10.1117/1.NPh.11.2.025005)
Supplement: Supplementary file 1 [file NPh_011_025005_SD001.pdf]

Supplementary Tables

Table S1 Temperature controller thermal optimization for 200 kHz line scan rate with a DC bias of 218 V and AC V_{pp} of 270 V

Power (mW)	Temperature set-point (°C)
10	31.20
50	31.04
100	30.96
150	30.84
200	30.76
250	30.70
300	30.58
350	30.48

Table S2 Optimized drive parameters versus drive frequency for the KTN-EOD

Line scan rate (kHz)	DC bias (V)	AC V_{pp} (V)	Temperature set-point (°C)
200	218	270	31.2
320	230	278	31.0
440	224	268	30.2
560	249	194	26.0
680	207	176	24.5
800	226	142	24.4
920	293	224	25.0
1040	276	134	26.0

Supplementary Figures

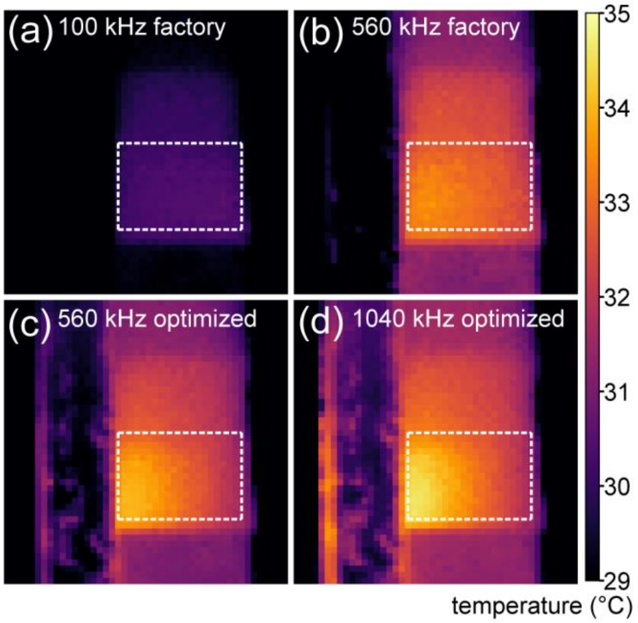

Fig. S1 Thermal images of the front facet of the KTN-EOD crystal under different drive conditions. KTN-EOD operated at factory DC bias, AC drive, and set temp and driven at (a) 100 kHz and (b) 560 kHz line rates. KTN-EOD operated at optimized DC bias, AC drive, and set temp [**Table S2**] and driven at (c) 560 kHz and (d) 1040 kHz line rates.
